# Design and Implementation of the Protein-Distinct Macronutrient-Equivalent Diet (PRODMED) Study: An Eighteen-Week Randomized Crossover Feeding Trial Among Free-Living Rural Older Adults

**DOI:** 10.1016/j.cdnut.2025.104588

**Published:** 2025-03-24

**Authors:** Bruna O de Vargas, Saba Vaezi, Jessica L Freeling, Yizi Zhang, Lee Weidauer, Chih-Ling Lee, Jing Zhao, Moul Dey

**Affiliations:** 1 School of Health and Human Sciences, South Dakota State University, Brookings, SD, United States; 2 FreelingBio Research Consulting, LLC, Vermillion, SD, United States; 3 School of Exercise and Nutritional Sciences, San Diego State University, San Diego, CA, United States

**Keywords:** RCT methodology, older adults, Midwestern, rural, controlled feeding, plant-forward, healthy eating pattern, animal protein, plant protein

## Abstract

**Background:**

Despite growing emphasis on plant-based eating, animal protein consumption remains high among Americans. The biological effects of different dietary protein sources within healthy plant-forward whole-diet patterns are poorly understood, and controlled-feeding methodologies for examining potential impacts are underreported.

**Objectives:**

This methods-report describes feasible approaches for menu planning and protein quality assessment within a 2-arm crossover randomized controlled feeding trial over 18 wk among rural Midwestern older adults. The primary trial aims to evaluate the effect of 2 protein-distinct diets on age-related health risk factors. The objectives of this methods-report are to *1*) describe the development of preportioned, ready-to-eat, macronutrient-matched, low ultra-processed, plant-forward, protein-distinct menus aligned with the Dietary Guidelines for Americans for home consumption, and *2*) present evidence-based strategies addressing the unique challenges posed by an all-food-provided, protein-distinct intervention.

**Methods:**

Participants completed 2 8-wk feeding phases separated by a 2-wk washout; one arm consisting of 162 g/d of lean pork (meat-protein source) and the other an equivalent amount of protein from pulses (plant-protein source). These primary proteins contributed ≥45% of protein intake within a 2000 kcal/d cyclic menu. Data quality, adherence, and participant experience were assessed using descriptive and inferential statistics.

**Results:**

Macronutrient distributions of the overall diets were closely matched despite differences in primary protein densities (meat-protein source 261.7 mg/g; plant-protein source 120.6 mg/g). Both diets featured increased fiber but reduced total and saturated fats, sodium, and ultra-processed foods compared to baseline. Consumed energy, though lower than provided, was matched between the intervention arms.

**Conclusions:**

Interventions were well-received, reflecting strong participant interest in healthier eating. Results illustrate a practical, scalable method for evaluating specific protein sources within a plant-forward diet, informing future studies and consumer practices.

The trials were registered at clinicaltrials.gov as NCT05577858 and NCT05581953.

## Introduction

Dietary protein *quantities* are associated with risk of sarcopenia and frailty in older adults, thus influencing independence in daily activities and the associated quality of life [[1], [2], [3], [4]]. However, there is limited knowledge about the wider biological implications of protein *quality* within comprehensive dietary patterns, particularly from well-designed controlled-feeding interventions and in relation to health risk factors in older adults. Research on satiety or health outcomes related to protein sources and quality has focused primarily on isolated protein supplements such as whey, soy, pea, and casein [[5], [6], [7], [8], [9], [10]]. Although supplementation strategies can be important to fill specific nutrient gaps, a whole-food approach is generally preferred to promote general health. Such an approach ensures a broad spectrum of nutrients in their most bioavailable forms in support of the body’s natural processes through synergistic effects. Epidemiological studies using the concept of a whole-diet matrix have reported conflicting evidence on the effects of protein quality and source on health outcomes. For example, in 2 studies, the substitution of as little as 3% of energy from animal protein with plant protein was inversely associated with cardiovascular disease and overall mortality [11,12]. In contrast, animal proteins have demonstrated superiority in delaying the onset of cognitive decline [13], and their enhanced anabolic properties suggest a role in preserving lean muscle mass during aging. To reconcile these, randomized controlled trial (RCT) based evidence regarding the impact of dietary protein type and quality on health is warranted. Such robust evidence may facilitate age-specific intake recommendations concerning protein type and quality. With the aging population rising in the United States and a growing interest in strategies to extend healthy lifespan (i.e., healthspan), the choice of protein sources within habitual diets may influence age-related health outcomes.

Protein quality is defined by the ability of a protein source to supply essential amino acids (EAA) in sufficient quantities and proportions, which are necessary for bodily functions such as protein synthesis and tissue maintenance [14,15]. However, the biological implications of the EAA ratios of dietary protein sources remain less studied in the context of dietary patterns and chronic health. Unlike some plant-based proteins, animal-based proteins contain the EAAs needed by humans in adequate amounts [[14], [15], [16]]. However, within the whole-diet matrix, the lower amounts of certain amino acids (AAs) in individual plant-protein sources are balanced out through protein complementarity, where different plant-protein sources are combined to compensate for lower concentrations of EAAs. The EAA content of an individual protein-rich food source, as well as that of the entire meal, contributes to the body’s anabolic response, which, over time, can influence metabolism, body composition, and overall health [[17], [18], [19], [20], [21], [22]].

Recently, plant-based diets have gained popularity in the United States [23,24]. The Dietary Guidelines for Americans (DGAs) align with a plant-based approach that does not necessarily exclude animal-source foods—which we refer to as plant-forward. DGA also encourages a shift away from excessive consumption of animal-sourced proteins, particularly red and processed meats, toward the inclusion of more lean proteins or plant-based options such as soy-based proteins, nuts, beans, peas, and lentils [25,26]. However, more Americans show a preference for animal-source foods, possibly due to taste, availability, ease of preparation, cultural traditions, general familiarity, or cost considerations because traditional animal proteins are often more affordable than many plant-based meat alternatives [27]. However, traditional plant proteins such as beans, lentils, and chickpeas, although currently less popular in the United States, are also more affordable than the industrial meat alternatives. Notably, healthy dietary patterns encourage whole, minimally processed plant foods over industrialized plant-based meat alternatives, which may contain higher concentrations of added salt, saturated fat, or other artificial ingredients. Despite not being the healthiest options, studies have shown that industrial plant-based meat alternatives are practical options to replace animal-source foods [28]. Whether evidence supports the shift from animal to plant-based proteins from the standpoint of nutritional adequacy, consumer acceptability, and health, however, remains less clear.

Pulses provide a plant-sourced protein alternative to soy. DGA allows pulses to be included as a protein source or as vegetables. The FAO of the United Nations defines the term “pulses” as edible seeds from the Leguminosae family [29]. Among the 11 recognized types of pulses, beans, peas, lentils, and chickpeas are more commonly consumed in the United States [30]. Pulse intervention studies using acute or partially controlled feeding have been reported [[31], [32], [33], [34], [35], [36], [37]]. However, a systematic review of these trials noted methodological concerns, including confounding factors, study design variability, limited generalizability due to health-impaired populations, and challenges in intake quantification due to self-reported data [38]. On the contrary, pork is a widely consumed animal-derived protein in the United States and globally. Our previous study elucidated the effects of daily pork consumption on the gut microbiota and microbiota-associated metabolites in comparison with white meat [39]. However, a side-by-side comparison of pork and pulse from the perspective of AA composition, nutrient adequacy, acceptability, and health outcomes remains unclear in the context of a healthy whole diet.

It is uncertain whether plant and animal protein foods can be considered nutritionally interchangeable. The DGA recommends protein substitution based on ounce equivalents [26]. However, proteins derived from plants and animals within a diet seldom match in terms of energy content, protein density, presence of other macronutrients, or EAA content, all of which influence the functional efficacy of protein-rich foods [40]. For example, an ounce equivalent of pork loin provides ∼7 g of total protein, whereas the same amount of cooked chickpeas offers approximately one-third that amount but comes packaged with fiber, which pork loin does not. Furthermore, a meal-matrix paradigm considers a significant contribution of the select protein source within the combined nutritional, metabolic, and physiological effects of all components in a meal rather than evaluating the protein sources alone without the context of the rest of the diet. This framework may help acknowledge the real-life scenario where the interactive effects of different nutrients and food matrices impact health outcomes. In a recent study that utilized this approach, it was shown that lean beef, compared to plant-based equivalents in a whole meal context, was more preferred and led to voluntary reductions in total carbohydrate and sugar intake in overweight middle-aged females, although this finding was based on a single day of *ad libitum* feeding [41]. Studies that incorporate prolonged feeding periods are essential to elucidate the broader implications of protein source exchanges across various dietary patterns and demographic groups. Such research will be crucial in refining protein intake recommendations.

In light of the limited number of feeding RCTs focusing on the effects of different protein sources in macronutrient-matched dietary interventions—especially in the context of a healthy dietary pattern such as the DGA—we propose a comprehensive methodology to tackle the specific challenges these studies face when conducted in a free-living setting. We discuss menu-planning challenges and mitigation steps undertaken while striving to *1*) provide adequate preportioned plant-forward meals that are acceptable, portable, and ready-to-eat to minimize variability in preparation methods and the need for consuming nonstudy foods and *2*) generate a practical and shareable meat or pulse-based eating plan that may be useful to both researchers and consumers. We also share the resulting protein quality differences in the overall diet, compliance, and the perceived study experience outcomes among the participants. Importantly, we report how the study diets improved the overall diet quality compared to the habitual Midwestern baseline dietary intake in the trial participants. The methods presented offer a valuable toolkit for future RCTs investigating the effects of dietary patterns and protein sources on health outcomes.

## Methods

### Overview and rationale

**FIGURE 1:**
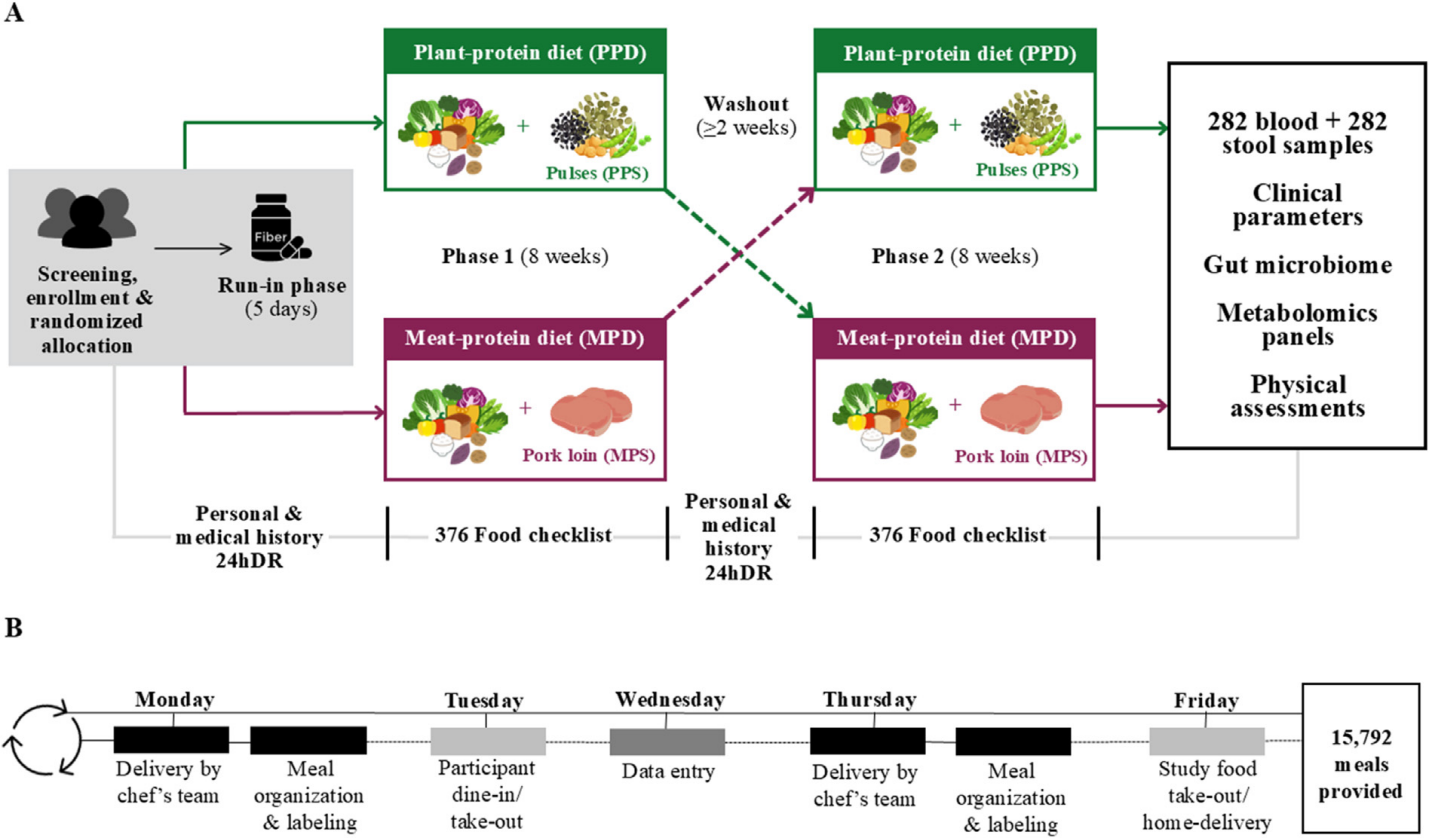
Overall study protocol (A) and weekly food service operations (B). MPS, Meat-protein source; PPS, Plant-protein source; 24hDR, 24-h dietary recall.

The core concepts of the protein-distinct macronutrient-equivalent diet (PRODMED) intervention methodologies described in this report will be used in the PRODMED1 and PRODMED2 trials, which were registered at clinicaltrials.gov (NCT05577858 and NCT05581953, respectively). The objectives, target sample size, and participant inclusion criteria of these parallel-running, overlapping trials are different. This report describes the dietary consumption and adherence data from the PRODMED1 trial in which 47 generally healthy males and females aged 60 y and older living in Brookings County of South Dakota completed the 2-arm feeding RCT conducted over 18 wk and submitted all required biological samples. The interventions contained 1 of 2 distinct protein-sourced diets for 8 consecutive weeks, followed by a 2-wk washout period before crossing over to the alternate protein source diet for an additional 8 wk (Figure 1A). For the washout, participants were instructed to return to habitual intakes. Inclusion and exclusion criteria for enrollment in PRODMED1 are described in Table 1. All study protocols followed the Declaration of Helsinki and were approved by the institutional review board (IRB) at South Dakota State University (IRB-2209011-EXP), and all participants signed informed consent forms. The primary objective of the trial was to compare the effects of plant and meat-sourced primary proteins on health risks within a DGA-macronutrient-aligned dietary pattern.

**TABLE 1:** Inclusion and exclusion criteria for enrollment of study participants.

Inclusion criteria	Exclusion criteria
Free-living adults aged ≥60 y, capable of and willing to drive to on-site study visits.	Major surgery or diagnoses (e.g., heart attack, cancer, stroke, etc.) within the past year.
Any race, ethnicity, and education level	Smoking or using recreational drugs.
Not underweight, body weight has been stable for the past 2 mo.	Special dietary needs (e.g., gluten-free, dairy-free) or picky eaters (menu discussed at screening).
Had a routine health checkup during the past year.	Currently, using medication or dietary supplements could affect study outcomes.
Agree to abstain from consuming alcoholic beverages, certain produce, and supplements during the 18-wk study period (list provided).	Existing diabetes (glycated hemoglobin check during screening visit) or known organ dysfunctions (e.g., hepatic and renal diseases)
Open to being assigned randomly to 1 of the 2 diet groups.	Significant gastrointestinal issues within the last 3 mo or known intolerance to a high-fiber diet.
Willing and able to provide written informed consent and follow study protocols.	Musculoskeletal conditions that may prevent physical fitness assessment.
Willing to inform the study director if and when antibiotic use becomes necessary, if there is any change in health status while on the intervention, or if any time conflicts with the study schedule.	Any mental health issue that could impair the ability to provide informed consent.
Willing to drink 64 oz of water or calorie-free fluids every day.	Plans to take a vacation during the intervention phases of the study.

To ensure treatment fidelity during the development of dietary interventions, emphasis was placed on creating protocols that maximized differentiation, defined as the degree to which the designed diets were sufficiently distinct to allow meaningful comparisons but maintained consistency. This consistency ensured uniformity across interventions in terms of nutrient intake, food preparation, serving methods, and approaches to support participants. Such a dual focus was crucial for the integrity of outcomes in the comparative analysis of the dietary interventions.

The comprehensive goals of the primary study presented unique challenges. Dietary studies exploring the impacts of protein on health often emphasize protein supplementation rather than a holistic dietary approach. Without careful control of the macronutrient distribution when comparing diet arms, the specific biological impacts of the protein source cannot be ascertained. To conduct a comprehensive and practical assessment of differential protein sources, this study adopted a whole-diet framework in free-living individuals provided with prepared meals. Diets were carefully matched for macronutrient distribution and fiber content within DGA recommendations. Notably, the menus were designed using foods readily available in local grocery stores and featured familiar items to ensure participant acceptance. To accommodate diverse taste preferences, menu options were carefully designed to avoid extreme flavors, promoting palatability while maintaining quality, variety, and portability.

Implementing controlled feeding over an extended duration in free-living contexts significantly enhances translational value by more accurately reflecting real-world conditions. However, this approach poses inherent logistical challenges for both researchers and participants. Participants are generally expected to consume only the foods provided by study staff [42]. The absence of personal choice in these studies, along with everyday temptations, can make adherence difficult for participants [43]. Challenges may include food cravings, allergies or aversions, social or travel-related disruptions, lack of motivation or perceived low benefits of compliance, and difficulty in avoiding habitual substances [44]. Additionally, factors such as study duration, cycling of menus, diet composition, and participant-researcher interactions play crucial roles in adherence.

Although these inherent logistical challenges are well-documented across controlled-feeding trials, the exploration of differential protein sources within a comprehensive dietary framework introduces unique secondary challenges. For instance, given that ounce equivalents of pork compared with pulses are not equal in protein density or vitamin B12 content, more eggs and dairy, pea protein powder, and commercially processed pulse-based meats were incorporated into the plant-protein diet (PPD). Similarly, because pork lacks fiber and some minerals compared to pulses, yeast flakes and soluble fiber were incorporated into meat-protein diet (MPD) menu items. Further, central to the primary RCT were longitudinal assessments of microbiome, physiological, and molecular health outcomes. These necessitated careful consideration of the impact of specific dietary components, such as indoles and fiber, which influence downstream metabolites of interest. Accordingly, adjustments to the experimental menu were essential.

In addition to aligning with the DGA macronutrient guidelines and being protein-distinct, the PRODMED goals also included meal preparation from scratch to reduce ultra-processed food (UPF) intake and an emphasis on a plant-forward approach that allowed dairy and eggs in both arms. Given these varied study objectives and the realities of the Midwest market supply and consumer pattern, we adapted the menus to incorporate certain concessions to enhance participant compliance. For example, we incorporated limited quantities of favorite foods being considered UPF, such as potato crisps and granola bars, into menus.

Despite the inherent logistical and study-specific challenges, the use of participant-experience surveys, diet quality assessments, and adherence evaluations has generated data supporting an achievable and systematic approach for developing robust menus for examining healthy, macronutrient equivalent, and protein-distinct interventions. Additionally, this methodological experience aligns with a previous study reporting a similar approach, describing RCT design, diet delivery, and adherence assessment [45]. Notably, the methodologies reported here offer clinicians and consumers a clear framework for how DGA-inspired, meat-based, and plant-based eating plans may be integrated into dietary practice to fit both omnivorous and vegetarian consumer preferences.

### Nutrient-matched menu design

The protein-distinct diets were designed to provide sufficient energy intake for weight maintenance, based on 2000 kcal/d using the 2020–2025 DGA for adults aged 51 and older. Both diets were matched for macronutrients (Supplemental Figure 1, Table 2). A 7-d cyclical menu was developed following the DGA nutrient recommendations (Supplemental Table 1A and B). Weekly supplementation with 5000 IU of vitamin D3 in both diet arms was necessary to meet 100% recommended dietary allowance (RDA) requirements.

**TABLE 2:** Nutritional summary of baseline intake and provided intervention diets compared to the 2020–2025 Dietary Guidelines for Americans.

Components	Recommendation	Baseline diet	PPD^1^	MPD^1^
Energy (kcal/d)	1600–2000	1957.1 ± 838.5	2021.8	2068.3
Protein [g/d (%E)]	10–35%	83.2 ± 41.5 (17.0)	86.8 (17.2)	93.2 (18.0)
Carbohydrate [g/d (%E)]	45–65%	211.3 ± 97.0 (43.2)	273.8 (54.2)	288.0 (55.7)
Fat [g/d (%E)]	20–35%	89.2 ± 55.1 (41.0)	68.9 (30.7)	67.6 (29.4)
Saturated Fat [g/d (%E)]	<10%	28.5 (13.1)	19.6 (8.7)	15.6 (6.8)
PUFA^1^ (g/d)	-	14.1	17.7	21.2
MUFA^1^ (g/d)	-	30.6	13.8	12.4
Total sugar [g/d (%E)]	-	82.1 (16.8)	69.5 (13.7)	74.7 (14.4)
Fiber (g/d)	22–28	20.2 ± 12.1	40.0	41.8
Sodium (mg/d)	<2300	2602.4 ± 1234.1	2194.9	2117.6
UPF^1^ (%E)	-	53.1	14.3	11.6
PF^1^ and MPF^1^ (%E)	-	46.9	85.7	88.4

Abbreviations: MPD, meat-protein diet; MPF, minimally processed food; MUFA, monounsaturated fatty acid; PF, processed food; PPD, plant-protein diet; PUFA, polyunsaturated fatty acid; SD, standard deviation; UPF, ultra-processed food.

1 Data represent mean ± SD. Macronutrient percentage distribution was calculated based on the total energy provided.

Meals prepared by a local chef (Chef Ellen, Inc) with experience in serving local consumers were delivered to the research laboratory twice weekly (Figure 1B). To ensure consistency and portion control, meals composed of fresh, high-quality ingredients were weighed out to the nearest gram and adhered to all food safety guidelines established during the study’s menu development process. This approach guarantees that all participants consumed uniformly prepared meals, maintaining consistency in food quantity, caloric intake, and nutrient composition while varying the protein source.

Two taste panels were held to determine the acceptability of the entrée items among adult Midwesterners and facilitate menu design. Several rounds of menu adjustments were made over 3 mo before the start of the feeding interventions. Cooking method adjustments were made based on participant feedback about the tenderness level of the pork, split peas, or seasonal produce to improve participant satisfaction. Seasonal weather changes in the upper Midwest affect the quality and availability of produce in grocery stores. For example, asparagus tenderness was compromised during the winter months and, based on participant feedback, was changed from fresh to canned. To ensure protocol uniformity, regular communication between the research team and the main chef took place throughout the study by meeting weekly in-person or via teleconference.

Participants received 3 meals, snacks, and select drinks free of charge for each day of the study. Each participant who completed the study received a total of 336 prepared meals, in addition to snacks and sides. A total of 15,792 meals were served to the 47 participants. The DGA macronutrient recommendations were followed while ensuring that adequate primary proteins were provided daily to account for ≥45% of the total protein in each diet across lunch and dinner (TABLE 2, TABLE 3). The snack options were adapted to ensure macronutrient balance based on primary protein source differences. The visual differences between protein sources hindered the blinding of diets.

**TABLE 3:** Protein quality of provided overall diet and primary protein sources.

Essential amino acid	Reference pattern mg/g protein	PPD	PPS	MPD	MPS
		mg AA/g protein	mg AA/g protein	mg AA/g protein	mg AA/g protein
Protein provided (g/d)	-	86.8	40.0	93.2	42.4
Histidine	16	20.5	24.5	27.3	43.2
Isoleucine	30	35.6	41.5	35.4	49.2
Leucine	61	61.1	70.8	61.5	85.2
Lysine	48	53.6	66.4	59.0	92.7
Methionine + cysteine	23	26.3	23.6	30.6	39.1
Phenylalanine + tyrosine	41	62.3	68.3	60.9	80.1
Threonine	25	28.8	30.2	32.8	44.9
Tryptophan	6.6	8.7	9.4	8.7	10.5
Valine	40	43.4	46.7	40.4	52.2
DIAAS^1^	≥100%	-	82	-	122.7
EAA-9 score^2^	≥100%	171	-	194	-

Abbreviations: AA, amino acid; DIAAS, digestible indispensable amino acid score; EAA-9, essential amino acid 9; FAO/WHO/UNU, Food and Agriculture Organization/World Health Organization/United Nations University; IAA, indispensable amino acid; MPD, meat-protein diet; MPS, meat-protein source; PPD, plant-protein diet; PPS, plant-protein source.

1 DIAAS was calculated using the FAO/WHO/UNU recommended indispensable amino acids scoring criteria. PPS DIAAS was determined using the mixture approach; when true ileal indispensable amino acid digestibility data were unavailable, true nitrogen digestibility was used. The IAA reference pattern was based on the adult age group (>18 y).

2 EAA-9 scores were calculated using the cumulative scoring framework following Forester et al. [52] (2023).

The MPD featured lean pork as the principal protein source (meat-protein source or MPS), whereas the PPD prioritized pulses (plant-protein source or PPS). This design effectively excluded other significant protein sources, such as soy and other animal meats, while incorporating grains, eggs, and dairy into both diets. The MPD was devoid of pulses (i.e., no chickpeas, red lentils, black beans, or dried split peas), and the PPD was devoid of any meats (i.e., no pork, chicken, beef, or fish). For the MPD group, the 162 g/d of cooked lean pork provided was within the range recommended by the Dietary Approaches to Stop Hypertension diet [46]. The primary protein in the MPD relied solely on lean roasted pork, which was entirely minimally processed and provided 261.7 mg/g protein density.

In contrast, the PPD group provided 331.6 g/d of pulse-based proteins with a combined density of 120.6 mg/g, which aligns with the daily pulse consumption amounts previously tested in clinical trials [38]. PPD included 7.8 g/d pea protein powder (Add A Scoop; Juice Bar Solutions, Inc), 32.1 g/d commercially processed pea protein-based plant meats (Beyond Meat, Inc), 52 g/d chickpea rice (Banza LLC), 61.9 g/d red lentil pasta (Barilla S.p.A), alongside 177.9 g/d of freshly cooked pulses (red lentils, chickpeas, split peas, and black beans) per person. Moderate inclusion of commercially produced ultra-processed plant meats in the PPD to mimic the texture of animal meat was opted for, thereby enhancing participant adherence while aligning with the protein source objectives.

### Menu design: plant-forward and high fiber

Both arms of the study included a plant-forward goal. The common perception toward increasing plant food consumption is that it helps reduce certain types of fat intake, such as saturated fats, while helping to increase fiber intake. However, for the sake of matching nutrients between the study arms, the menu strived to match these components as closely as possible. Participants in both diet arms were provided with 14 or more daily servings of plant foods averaged from the weekly menu cycle, including vegetables, whole grains, and fruits. Pulses were classified within the protein category. Breakfast, lunch, and dinner provided plant-based foods at 3 main meals every day, keeping UPFs below 13% of total calories, respectively (Supplemental Figure 1A and B, Box 1, Table 2). Both MPD and PPD intervention diets contained higher fiber than the habitual baseline diet (Table 2), and thus, participants were adapted to the higher fiber content before the start of the 18-wk intervention during a 5-d run-in period. To achieve this, participants were provided with 2 prebiotic fiber supplements daily for 5 d, with each chewable tablet containing 2 g of fiber, resulting in a total daily intake of 4 g (Equate-brand). Soluble fiber powder (Equate-brand, wheat dextrin) was added to the MPD recipes to match with PPD. Participants were asked to avoid additional fiber supplementation during the study.

BOX 1 **Features of plant-forward intervention diets**

**Table:** 

Weekly basis
• Plant food at every meal and a high-fiber diet
• Vegetables, whole grains, and fruits ≥102 servings
• Dairy and eggs ≥12 servings (in moderation to maintain primary protein dominance)
• Nuts and seeds ≥0.78 servings (minimal to maintain primary protein dominance)
• Spices ≥66g
• Plant oil ≥8 servings
• Shelf-stable ultra-processed snacks allowed in moderation, ∼9 servings
• No soy, beef, chicken, seafood, games (to avoid conflict with primary protein choices)
• No artificial sweeteners in recipes
• Emphasis on fresh ingredients and reduced processing
• Plenty of calorie-free fluid
• No alcohol

Alt-text: BOX 1

### Menu design: diet-alignment and compliance

Participants were provided with ∼2000 kcal/d and encouraged to eat to their comfort level, as caloric restriction was not a study goal. Provisions were included for adjustment of caloric intake for potential weight gain; however, no such event was recorded across any cohort. For higher calorie needs, additional portions of study foods were provided on a one-on-one basis that aligned with their assigned dietary intervention. Participants were instructed to refrain from alcohol, antibiotics, certain supplements, fiber supplements, probiotics, outside food, and smoking for 5 d before and during each feeding phase. Once the enrolled participants started the intervention, communication was ≥2 times weekly between participants and research staff to reinforce motivation and compliance.

Daily food intake was tracked to limit outside food consumption. Importantly, certain foods that matched the dietary plan were not classified as noncompliant but counted toward actual intakes. Noncompliance was defined by the ingestion of high-calorie and specific off-limit items or those containing UPF. Examples of noncompliant foods include creamer, heavy cream, ice cream, candy, sugar-sweetened beverages, donuts, cookies, pizza, birthday cakes, and similar foods that were not permitted within the dietary framework. Thus, any deviation from the assigned diet, particularly involving UPF, was deemed a breach of compliance.

To illustrate, both study arms aimed for a plant-forward, high-fiber diet low in UPF. Therefore, enjoying extra garden vegetables, such as carrots or cucumbers, was considered outside food but study-aligned and thus compliant. Although small portions of some popular UPFs, such as potato crisps and granola bars, were provided as part of the study to help reduce attrition, these were limited (Supplemental Table 1A and B). Therefore, any extra consumption of packaged food such as potato crisps, regardless of the study arm, would be considered noncompliance due to the lower UPF target. These approaches not only enabled accurate data collection but also fostered a positive participant experience.

Furthermore, compliance was also study-arm specific based on protein source. For example, the consumption of pork during the PPD phase constituted a compliance breach, as did the intake of pulses during the MPD phase. Participants in the MPD phase reporting substitutions such as bacon and ham for pork were deemed noncompliant due to their highly processed nature, in contrast to the minimally processed products provided in the study.

Unique participant preferences were revealed in this rural study population, underscoring the need for flexibility in study design to enhance retention. As described, food service was conducted twice weekly, necessitating frozen, cupped, or dried fruits. However, participants expressed a distinct preference for fresh fruits, a sentiment bolstered by a common interest in gardening in this age group. In response to this feedback, substitutions were allowed. Participants could replace some provided items with fresh produce upon request, thereby fostering compliance and satisfaction. We did not consider this noncompliance and accommodated it by removing specific items from provided meals, enabling them to substitute their own fresh alternatives upon request.

Many participants reported habitual consumption of artificially sweetened beverages, expressing concern about their ability to adhere to the study recommendation of consuming 64 oz of plain water, black coffee, or black tea over the study duration. Thus, although artificial sweeteners were excluded from recipes, participants were permitted to consume calorie-free, artificially sweetened beverages in moderation, defined as 2–3 12-oz servings per week and ≤1 5-calorie drink packet (e.g., Crystal Light) per day. Further, 0-calorie flavored syrups were made available upon request to those who preferred an alternative to plain yogurt.

Finally, due to the Thanksgiving Holiday and the common practice of social gatherings involving large meals in the Midwest, accommodation was made for participants to engage in 1-holiday meal. Although participants were permitted to consume a nonstudy-provided meal, they were instructed that the meal must be recorded and aligned with their assigned study arm regarding protein source. Alternatively, participants had the option to obtain a favorite study-provided meal. This meal, whether provided or outside, was recorded according to the compliance criteria outlined above.

### Menu design: biological sampling

The intestinal microbiome and AA metabolites are critical components of the primary RCT experimental paradigms. Indole-containing foods, particularly cruciferous vegetables, are known to influence compounds such as trimethylamine N-oxide production [47]. Accordingly, during the in-person screening, participants were provided with a list of specific foods that could adversely affect biological sampling results. They were instructed to avoid these foods for 48 h prior to the collection of blood, urine, and stool before entering an intervention phase. The excluded foods encompassed a range of indole-containing cruciferous vegetables, including arugula, bok choi, broccoli, brussels sprouts, cabbage, cauliflower, collard greens, horseradish, kale, kohlrabi, mustard greens, mizuna, napa cabbage, radishes, rutabaga, tatsoi, turnips, wasabi, and watercress as well as grapefruit. Additionally, trimethylamine N-oxide-containing foods such as fish and seafood were excluded. Although the study diets may have contained some of these foods, measures were implemented to either exclude or distribute them evenly to mitigate their potential confounding on blood results. For instance, although broccoli was incorporated into the study diets in an even manner, it was restricted during the run-in phase to prevent uneven interference with baseline blood marker results. Furthermore, participants were advised against the use of pain relievers within 24 h prior to blood collection to minimize potential alterations in inflammatory biomarkers.

### Menu: food safety

All personnel involved in food handling were required to possess current ServSafe certification (National Restaurant Association Educational Foundation, Washington, DC, United States) in addition to having completed the Collaborative Institutional Training Initiative Program (Fort Lauderdale, FL, United States) training in human subject research and good clinical practice. This ensured adherence to the highest food safety and clinical research standards. Single-serve study foods were prepackaged for grab-and-go convenience in single-use, Bisphenol A-free, freezer-, microwave-, and oven-safe containers. The chef’s team sourced fresh ingredients from local grocery stores (Walmart Inc and Hy-Vee Inc), mimicking consumer access. Food preparation following strict sanitation protocols occurred in a licensed commercial kitchen (Chef Ellen’s Kitchen, Sioux Falls, SD, United States), and meals were delivered to the Human Feeding Laboratory at South Dakota State University (Brookings, SD, United States) for storage, labeling, and distribution by trained staff. Meals were stored in commercial refrigerators and freezers with stainless steel exteriors and interiors, maintaining temperatures between 1°C and 4°C for refrigerators and –18°C for freezers. Meals were stored refrigerated or frozen until pick-up by participants or heated for participant dine-in at the food service station within the Human Feeding Laboratory. Participants received detailed instructions on the safe storage and reheating of prepackaged meals (Supplemental Box 1).

### Dietary content analysis

Although nutritional analyses in feeding studies commonly rely on software linked to food composition databases due to practicality, these tools often provide incomplete information on certain food components, particularly those from commercial sources. This limitation can affect the accuracy of dietary assessments [48]. To enhance precision, manufacturers were contacted to obtain AA profiles for select protein source foods, such as lentil pasta. In addition, a composite analysis of the intervention diets was carried out. All items from the weekly menu were combined proportionately and homogenized into a paste. The paste was freeze-dried to obtain its moisture content. Protein contents in the dried samples were analyzed based on the Dumas method using an Elementar Rapid N Exceed nitrogen analyzer (Elementar Americas Inc). Aspartic acid was used as standard. A conversion factor of 6.25 was used to calculate the protein content (percentage) in the dried samples. The protein content (grams/day) in the diet was calculated by adjusting for the moisture content and serving size.

To gain a deeper understanding of food intake in terms of processing, the NOVA classification system was used to identify and estimate the intake of UPF through the categorization of foodstuffs into 4 groups. Foods were classified based on NOVA and food compass nutrient profiling systems as either UPF or grouped as processed or minimally processed. Foods were manually classified as *1*) unprocessed or minimally processed, *2*) processed culinary ingredients, *3*) processed, or *4*) ultra-processed [49]. For certain ambiguous food items, the studies by Mendonça et al. [50] (2016) and Valmorbida et al. [51] (2023) served as helpful references. Using the NOVA system, we generated an estimate for the percentage of energy contribution of UPF of each study-arm diet.

In addition to overall diet quality, the overall protein quality of the PPD and MPD was evaluated using the EAA 9 score proposed following Forester et al. [52] (2023). This score ensures that dietary recommendations for each EAA are achieved within a whole-diet context. The EAA-9 score is derived from the minimum percentage of the RDA fulfilled by each AA, ultimately reflecting the lowest percentage adequacy achieved by any individual essential AA. A diet is deemed to fulfill EAA RDAs when the EAA-9 score reaches 100%.

Primary protein quality was assessed using a digestible indispensable AA score (DIAAS). DIAAS offers a refined approach by directly measuring the digestibility of individual AAs in the small intestine, utilizing data from swine and, occasionally, humans. DIAAS was calculated following the FAO/WHO/UNU recommended indispensable AA scoring criteria. The plant-protein source DIAAS was determined using the mixture approach, wherein true ileal indispensable AA digestibility values were used when available; otherwise, true nitrogen digestibility was applied. The indispensable AA reference pattern was based on the adult age group (>18 y). DIAAS scores can exceed 100%, indicating a superior quality of protein [15]**.**

### Participants: enrollment and run-in

The intervention was conducted between 24 January 2023 and 19 December 2023 on a rolling-enrollment basis. Participants were recruited via IRB-approved flyers and advertisements announced on bulletin boards throughout the community and in a local newspaper between December 2022 and July 2023. Additionally, outreach efforts included broader distribution via Every Door Direct Mail and targeted emails sent to previous trial participants. Research staff conducted a ∼20-min telephone screening with interested individuals, providing an overview of the intervention and assessing medical histories, lifestyle habits, and willingness to participate in the feeding trial. Eligible individuals aged 60 y or older and in general good health were invited for laboratory-based screening to confirm eligibility. Comprehensive inclusion and exclusion criteria are detailed in Table 1.

During the initial in-person visit, individuals were given extensive information regarding the study’s objectives, procedures, potential risks, and anticipated benefits. Eligibility was evaluated through a questionnaire addressing personal and medical history, alongside measurements of height, weight, and glycated hemoglobin concentrations. A personal and medical history questionnaire was obtained both during the initial in-person screening visit and at the beginning of the second phase, after the 2-wk washout (Figure 1A). The questionnaire gathered demographic information, including age, sex, race, marital status, and educational background, as well as past and present health conditions, medication and supplement usage, sleep duration, and daily physical activity levels.

### Participants: meal engagement

The meal pick-up for participants was scheduled on Tuesdays and Fridays, with dine-in options exclusively available on Tuesdays, subject to specific circumstances on Fridays. On Tuesdays, breakfast meals were prepared and served upon the participant’s arrival, accompanied by freshly brewed coffee (Figure 1B). If blood collection was required, the sample was collected in the fasted state prior to the meal. Participants presented their menu checklist and food intake entries from the prior week, which could be different from what was provided. Brief explanations of any necessary instructions were provided, and participants were asked if they required supplemental items such as extra portions, salt, pepper, sweeteners, or flavorings. Assistance was offered to those who needed help transporting their meal bags to their vehicles. In exceptional cases, home delivery by research personnel was available for participants unable to collect their meals in-person. Postdistribution, the area was maintained in a clean and organized manner, including the collection of dirty dishes for washing. A comprehensive quaternary cleaning of the area was conducted immediately following each session.

In addition to the regularly scheduled interactions between participants and research staff at dine-in and pick-up, participants had 24/7 access to both the research team and the principal investigator via text and email. Communication was frequent during holidays and after hours, as unexpected situations could arise. Several participants traveled during the feeding phases and carried frozen meals with them to avoid consuming outside food.

### Participants: dietary intake assessments

Dietary intake, reflecting both recent and habitual consumption, was evaluated through a 24-h dietary recall administered prior to the onset of each intervention phase (Figure 1A). In addition, study staff recorded participants’ intake of alcoholic beverages, supplements, antibiotics, grapefruit, and food from the Brassicaceae family. Participants then documented their own food consumption over the preceding 24 h, specifying portion sizes and food preparation methods. A WebMD portion size guide was provided to the participants.

To understand participant experiences during each dietary intervention phase, we designed a participant-experience survey. The survey was administered after each 8-wk intervention phase. Participants evaluated several factors, including hunger, fullness, overall diet satisfaction, convenience of eating, food quality, and fiber-related gastrointestinal issues.

During the study, participant adherence to the designated interventions was systematically monitored through the use of a daily food checklist. This checklist included a detailed list of meals and food items provided, allowing subjects to document any food not consumed, additional food requests made to staff, and any outside food items ingested. Checklists were collected during meal pick-ups, scheduled twice weekly. The checklist information was entered into Nutritionist Pro (Axxya Systems LLC) for data analysis.

### Participants: biological assessments

Anthropometric and muscular fitness measurements were obtained from participants at baseline/pre-diet and on a postdiet basis. Dual-energy x-ray absorptiometry for body composition was utilized at baseline and post-diets to quantify lean mass and fat mass and estimate visceral, android, and gynoid fats (Hologic Horizon). Height was measured in duplicate, without shoes, using a portable stadiometer (Seca), and weight was recorded in duplicate on a digital scale (Model 770; Seca) with light clothing; mean values were used for analysis. Blood pressure was assessed using a GE Carescape V100 vital signs monitor (General Electric) after participants had been seated for a minimum of 5 min. Physical activity levels were estimated using a pedometer (Neskla), worn for 3 d; measurements were repeated at the beginning of each phase, and the mean daily step counts were logged. Participants were instructed to maintain their typical daily activity levels throughout the study, which varied from sedentary to highly active. A trained phlebotomist collected venous blood samples from participants following an overnight fast at baseline and at weeks 4, 8, 10, 14, and 18 (end of controlled feeding). Each collection included 3 10-mL heparin-coated (green) plasma tubes and 1 10-mL serum separator (red) tube. Plasma and serum samples were aliquot and stored at –80°C for subsequent analysis of primary and secondary outcomes, including metabolites, and biomarkers. Two hundred eight-two blood and stool samples were collected at 6-time points from 47 participants who completed all study requirements.

For stool and urine collection, participants received a stool kit (Omnigut stabilizer; DNA Genotek Inc), as well as instructions for collecting first-morning urine at home. Stool and urine samples were preserved for future assessments.

### Calculations and statistics

The diets were designed, calculated, and analyzed using Nutritionist Pro Software (version 8.1.0, Axxya Systems), with comprehensive menus detailed in Supplemental Table 1A and B. For the RCT, participants were assigned to intervention groups through a 2 x 2 block randomization method, using blocks of 2 for individuals and 4 for couples. This allocation process was conducted in a blind manner by a researcher not involved in future data analysis. The data are presented as mean ± SD or *n* (percent), with all values undergoing double validation. All data were analyzed, and visualizations were created using RStudio version 4.4.0. Normality was assessed via the Shapiro-Wilk test. Paired t-test or Wilcoxon signed-rank test with continuity correction was utilized to assess diet compliance outcomes. Statistical significance was established at *P* ≤ 0.05.

## Results

### Total energy

The intervention diets provided were well matched for the total energy (Table 2). The total consumed energy was also close between the arms (1605 ± 364 kcal/d, PPD, 1695 ± 320 kcal/d, MPD) but was lower than both the provided average 2000 kcal/d and that of the baseline (1957 ± 838 kcal/d) intakes (Table 4) resulting in weight loss (data not shown). Participants were advised to maintain their habitual activity levels throughout the study, and step count data were collected to confirm (data not shown). A similar level of voluntary calorie reduction in both arms may be attributed to the controlled crossover design of the study. However, *ad libitum* intake allowed by the study design led to an average 90 kcal/d difference between the 2 diet arms (*P* < 0.05).

**TABLE 4:** Estimated actual intake of the intervention diets as a measure of compliance.

Components	PPD	MPD	*P* value
Average energy provided per day (kcal)	2021.8	2068.3	-
Study foods not consumed (kcal/d)^1^	492.1 ± 341.1	471.4 ± 319.9	0.274
Protein (g/d)	21.7 ± 15.6	22.1 ± 15.6	0.698
Carbohydrate (g/d)	66.5 ± 46.6	66.0 ± 45.0	0.826
Fat (g/d)	16.6 ± 11.2	15.0 ± 10.0	0.011
Fiber (g/d)	10.5 ± 7.5	9.7 ± 6.9	0.121
Extra study foods consumed (kcal/d)^1^	45.0 ± 82.3	47.7 ± 65.2	0.518
Protein (g/d)	1.5 ± 2.7	1.5 ± 2.2	0.712
Carbohydrate (g/d)	6.3 ± 12.6	6.6 ± 9.3	0.582
Fat (g/d)	1.6 ± 2.5	1.7 ± 2.4	0.510
Fiber (g/d)	0.6 ± 1.3	0.6 ± 1	0.831
Nonstudy foods consumed^3^ (kcal/d)	30.7 ± 42.9	50.5 ± 83	0.066
Protein (g/d)	0.8 ± 1.1	1.4 ± 2.3	0.008
Carbohydrate (g/d)	4.2 ± 5.4	6.3 ± 8.7	0.096
Fat (g/d)	1.3 ± 2.4	2.4 ± 5.1	0.085
Fiber (g/d)	0.6 ± 0.8	0.8 ± 1.3	0.062
Net energy consumed (kcal/d)^2^	1605.4 ± 364.1	1695.1 ± 320.1	<0.001
Protein (%E)	16.4 ± 0.7	16.9 ± 0.9	<0.001
Primary protein (%E)	7.0 ± 0.7	7.0 ± 1.0	0.904
Carbohydrate (%E)	53.1 ± 1.1	53.8 ± 1.2	<0.001
Fat (%E)	30.5 ± 1.1	29.3 ± 1.5	<0.001
Fiber (g/d)	31.7 ± 7.7	33.6 ± 7.2	<0.001
Sodium (mg/d)	1674.8 ± 389.8	1688.4 ± 337.4	0.466

Data presented as mean ± SD. *P* values were derived from paired *t*-tests when comparing PPD and MPD compliance for normally distributed data. For nonnormally distributed data, *P* values were derived from the Wilcoxon signed-rank test with continuity correction.

Abbreviations: MPD, Meat-protein diet; PPD, Plant-protein diet; SD, standard deviation.

1 Caloric restriction was not a goal. Participants could consume all or part of the food provided based on their comfort. When needed, they could request additional portions.

2 Actual food intake (net energy consumed) = standard provided food + extra study food (if any) + nonstudy food – nonconsumed study food.

3 Nonstudy food was considered noncompliance.

The contribution of nonstudy foods to the overall average energy intake was negligible and similar between groups, with PPD contributing 2% and MPD contributing 3% (*P* > 0.05). Moreover, 41% and 33% of calories from these nonstudy-provided foods were aligned with PPD and MPD study-arm definitions, respectively, indicating reasonable adherence to plant-forward eating principles. The observed decrease in total energy consumption correlated with a proportionate reduction in nutrient intakes in both groups. Although both groups exceeded DGA fiber recommendations, there was a 1.9 g/d difference in fiber intakes between PPD and MPD (*P* < 0.001). (Table 4). A 50% increased fiber consumption, from an average of 20 g/d at baseline to over 30 g/d during the interventions, was observed.

### Macronutrients

**FIGURE 2:**
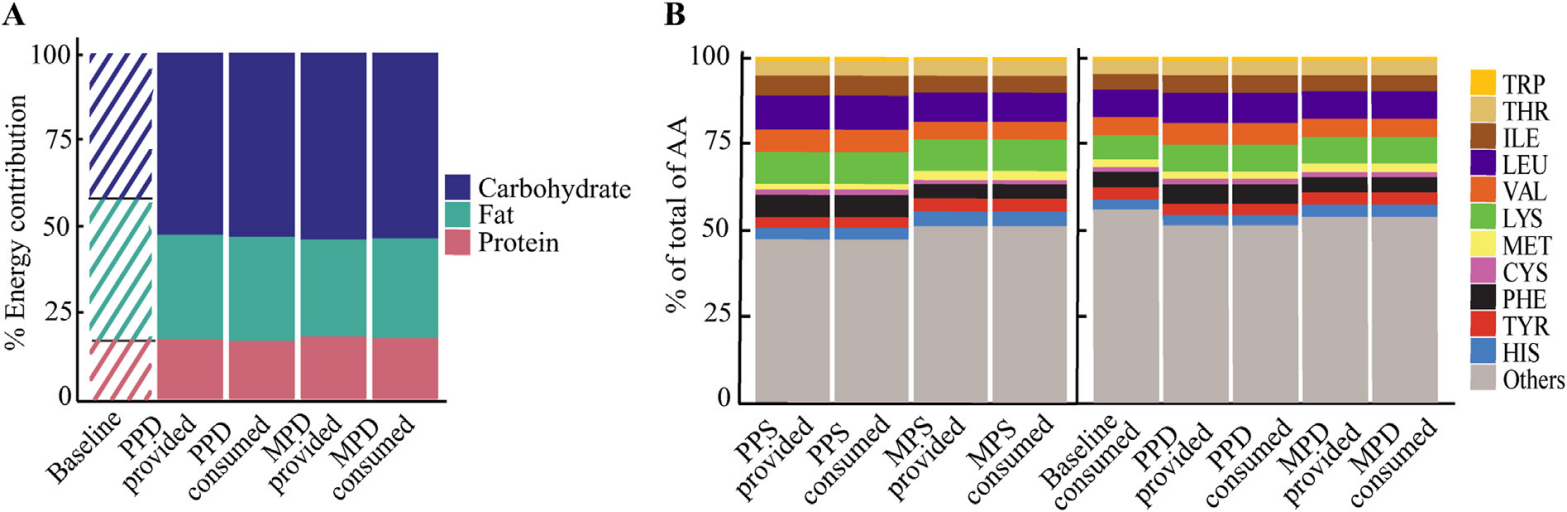
Distribution of provided and consumed macronutrients at baseline and across intervention arms (A), comparison of essential and semi-essential amino acids—provided compared with consumed (B). AA, amino acid; CYS, cysteine; HIS, histidine; ILE, isoleucine; LEU, leucine; LYS, lysine; MET, methionine; MPD, meat-protein diet; MPS, meat-protein source; PHE, phenylalanine; PPD, plant-protein diet; PPS, plant-protein source; THR, threonine; TRP, tryptophan; TYR, tyrosine; VAL, valine.

Provided intervention diets were higher in carbohydrate (+12%) and lower in fat (–11%) than baseline intakes. The macronutrient ratios of the consumed calories in the 2 intervention groups were similar and followed the patterns of the provided PPD and MPD (Figure 2A**,** Table 2). Although the percentages of total fats in both PPD and MPD were well matched, with ∼30% of calories coming from fat, the 2 diets exhibited slightly different relationships among fat types. The PPD diet provided slightly higher SFAs and MUFAs but slightly lower PUFAs compared to the MPD (Table 2). Both intervention diets demonstrated a higher carbohydrate content compared to baseline, with comparable levels between PPD and MPD (*P* > 0.05). Reductions in total sugars were noted between baseline and intervention diets (Table 2).

There were minimal differences (all, 17–18% of total energy) between the average protein intake levels at baseline (83.2 g/d) and the provided diets (86.8 g/d in PPD and 93.2 g/d in MPD). Protein content analyzed using Nutritionist Pro closely aligned with composite analysis for total nitrogen content in the diet (87.47 g/d in PPD and 93.6 g/d in MPD). Both diets provided high EAA-9 scores (PPD 171, MPD 194) that were slightly higher than baseline and exceeded the RDA recommendations for EAA; however, there were differences in EAA and nonessential AA composition as well as in DIAAS score of the primary proteins (Figure 2B, Supplemental Table 2, Table 3).

### Baseline compared with provided diet quality

The quality of the baseline diet provided an important snapshot of the Midwestern habitual diet in an older adult age group. The EAA-9 score was >100 at baseline (158%, data not shown), suggesting this population’s protein quality intake was more than adequate. NOVA analysis suggested a high intake of UPF [53], contributing 53% of total calorie intake at baseline (Table 2). However, this UPF intake was slightly lower than the 2009–2010 NHANES data, which reported that UPF contributed 58% of total caloric intake in the general population [54]. Both intervention diets decreased UPF %energy (%E) similarly and substantially (–38.8%E, PPD, –41.5%E, MPD). Compared to baseline intakes, the study diets provided lower amounts of total (–10.3%E, PPD, –11.6%E, MPD) and saturated (–4.4%E PPD, –6.3%E MPD) fats, sodium (–407.5 mg/d, PPD, –484.8 mg/d, MPD), and total sugars (–3.1%E PPD, –2.4%E MPD), whereas significantly increasing fiber from 20 g/d to over 40 g/d (Table 2).

### Menu acceptability and adherence

**FIGURE 3:**
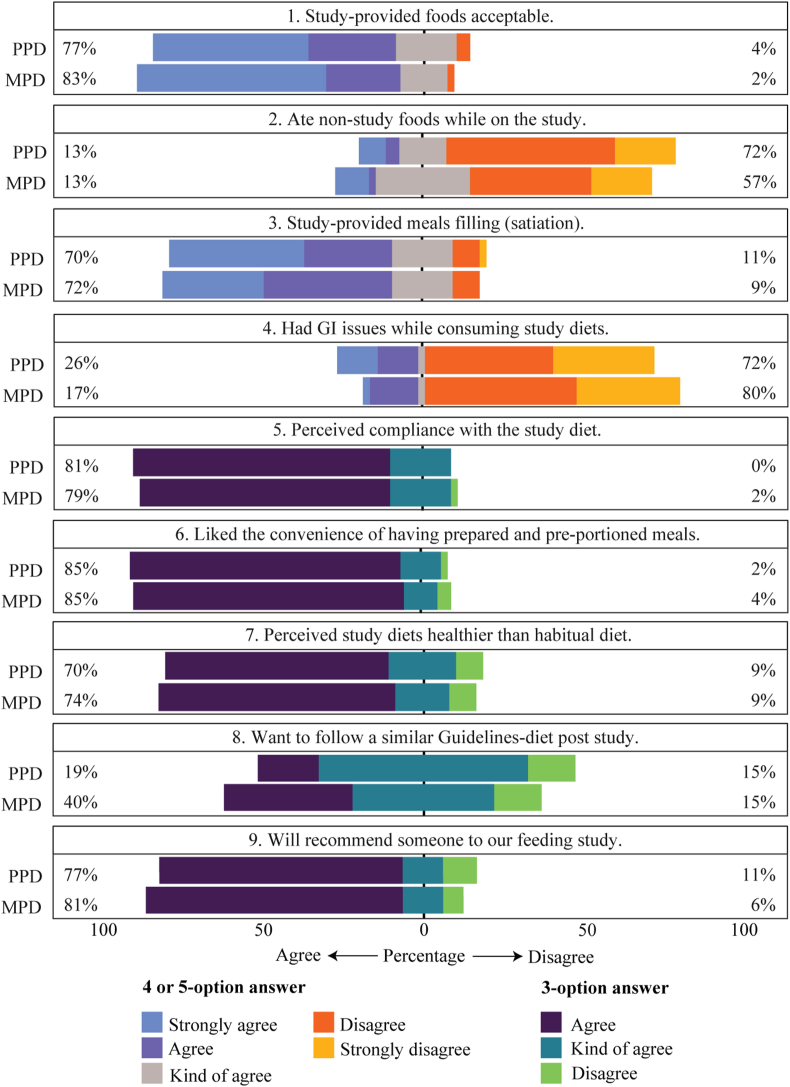
Scores from the study experience survey. MPD, meat-protein diet; PPD, plant-protein diet; GI, gastrointestinal.

Although participants found study-provided MPD foods slightly more acceptable than PPD at 83% compared with 77%, respectively (Figure 3, Question 1), >70% of both groups found the study-provided meals filling (Figure 3, Question 3). Just 13% of participants in both PPD and MPD admitted that they ate nonstudy foods during the intervention phases of the study (Figure 3, Question 2), although the *nonstudy foods* perceived by participants may include some study-aligned foods like garden vegetables that were otherwise not provided by the researchers. About 80% of participants in both diet arms believed they were compliant with the study diets (Figure 3, Question 5), and 85% of participants in both groups liked the convenience of ready-to-eat meals (Figure 3, Question 6). Although ≥76% of participants were willing to recommend the study to others (Figure 3, Question 9) and 70% perceived the study diets as healthier than their habitual diets (Figure 3, Question 7), responses were mixed about long-term use. Specifically, 19% and 40% of PPD and MPD participants, respectively, indicated they would want to follow a similar dietary lifestyle after the study (Figure 3, Question 8). Finally, 72% of PPD and 81% of MPD reported no or minor gastrointestinal issues while consuming intervention diets (Figure 3, Question 4) despite consuming 50% higher fiber than baseline intakes. A summary of key considerations when designing meal provision ensuring greater compliance in randomized feeding-controlled trials is provided (Supplemental Table 3).

### Attrition

The study recruitment flyer contained eligibility information, and as a result, 78 of the 106 interested participants passed the phone screening and were scheduled for informed consent signing, on-site screening, enrollment, and randomized allocation to a study arm. During the 5-d run-in phase that followed this initial enrollment, 17 participants withdrew, primarily citing personal reasons or unforeseen scheduling conflicts. Once the participants began the intervention, the dropout rate was 20.3%. A total of 6 participants, 3 in each intervention group, did not like the study diets and dropped out during the first diet phase, with an additional 6 participants dropping out after that due to health issues unrelated to the diets (e.g., pneumonia, COVID-19, etc.) or other personal reasons.

## Discussion

This methods-report summarizes the efforts to plan, design, implement, and monitor adherence to 2 plant-forward diets aligned with the 2020–2025 DGA guidelines but differing in the primary protein source. We evaluated protein quality differences while macronutrient ratios matched in provided and consumed diets, both differing, however, from Midwestern habitual intake. The report details comprehensive methods designed for a one-to-one comparison of distinct protein sources within a healthy whole-diet format using a tightly controlled and ready-to-eat all-food-provided format. The menus for both diets used fresh, canned, and frozen foods to provide diets that a rural United States population could realistically achieve beyond a study setting. The menus were consistent from week to week and between the intervention groups, except for select menu options that compensated for the differences in the total protein and fiber content provided by meat compared with plants. The intervention diets were markedly lower in UPF compared to the habitual baseline diet, which itself was marginally lower than the average national UPF consumption. Macronutrient distributions matched the intervention groups for both the provided diets and the actual intakes, highlighting the effectiveness of controlled feeding within a crossover design where participants serve as their own controls. The older adult participants in this study represent a rapidly growing segment of the United States population at risk of a reduced health span, with the potential to benefit significantly from dietary improvements that enhance their health-related quality of life.

### AA Variation studies

There has been a growing interest in the biological activities of specific AAs in relation to cardiometabolic risks, as well as cognitive and muscular health. However, most existing research has used animal models or observational designs in humans, often neglecting to adjust for total protein intake [19,[55], [56], [57], [58]]. Furthermore, much of this research used supplementation or formula diets, which are logistically simpler to conduct compared to an all-food-provided approach that mimics realistic food exposure [59,60]. Also, studies report the effects of protein quality variations when protein intake quantities are below or above the recommended intake ranges for healthy humans, making the outcomes less relatable to the general population [61]. In this protein quantity-matched trial, the tested protein sources were the primary contributors of protein to the diet, and both diets exceeded the 100% EAA adequacy requirements, thus controlling for potential confounding factors related to protein deficiency.

### Adherence strategies in an older free-living population

Randomized controlled-feeding trials are characterized by high costs and significant demand on researcher effort, but if well executed, they can provide strong, definitive evidence in humans [62,63]. However, achieving high levels of adherence to behavioral or lifestyle interventions can be particularly challenging, especially in real-world environments [64]. Dietary feeding RCTs present greater logistical challenges compared to RCTs involving pharmaceutical, nutraceutical, or dietary supplements. This complexity arises from the nature of whole-diet interventions, which depend significantly on human behavior and subjective experiences. Consequently, achieving standardized dosing (e.g., primary protein intake) and maintaining precise control over administration are hindered by the inherent variability in food intake, preparation methods, and adherence levels. Moreover, no established standard currently exists for effectively monitoring adherence in free-living controlled-feeding trials.

To address these logistical challenges, participants were provided with prepared, preportioned, ready-to-heat meals and adherence checklists to record intakes at each meal. Nevertheless, the burden on participants remained high compared to domiciled studies, where participants lived or ate solely at the research center. This study required participants to dine-in or pick-up meals twice weekly, which created a challenge for some participants but also fostered interaction with researchers and improved compliance. Contracting with a local chef reduced the burden of meal planning, preparation, and grocery shopping while ensuring culturally relevant, accessible meals aligned with participants’ dietary habits and preferences, though it required increased coordination with the research staff. Compared to in-house food preparation by trained staff, which we employed in our prior studies, both approaches are viable and adaptable to feeding studies requiring rigorous protocol fidelity. Each single-serve meal box was labeled with participant ID, meal information, and heating instructions, minimizing confusion and facilitating compliance. Label colors were distinct for breakfast, lunch, and dinner entrées for easy identification by participants. Real-time feedback during food pick-ups and daily food adherence checklists enabled the research team to mitigate noncompliance and adapt menus quickly when needed.

We employed cyclical menus, which are logistically ideal for dietary interventions in free-living individuals as they streamline menu planning and preparation. Cyclical menus can sometimes reduce adherence due to meal repetition and potential participant fatigue. However, participant-experience surveys highlighted the acceptability of the intervention, with participants reporting the meals as convenient and satiating and expressing a willingness to recommend the study to others. These findings indicate that cyclical menus, despite their potential drawbacks, did not appear to negatively impact adherence in this study. However, cyclical menus may become a concern in longer interventions.

The dedication to study compliance was notably high among the study participants. Many individuals were returning as volunteers from past studies in this small Midwestern town and were familiar with the demands of controlled-feeding trials. Their willingness to return, coupled with numerous requests for copies of study recipes at the trial’s conclusion, underscores strong consumer interest in healthy dietary patterns and participating in feeding studies. The real-world setting may have yielded more relevant data, particularly given the rural demographic. The principle of “aging in place” and maintaining independence among older adults also suggests a preference for participation in studies that allow at-home living [65]. These insights underscore the importance of designing studies that balance participant burden with the need for relevant and generalizable findings. Controlled-feeding studies, particularly in older adults, remain underreported compared to observational nutrition studies. These findings collectively highlight the feasibility of implementing low-UPF, plant-forward, and protein-distinct dietary interventions within free-living aging populations—an important but often overlooked demographic.

### Population-specific and consumer insights

Certainly, some practical challenges were presented in this population of older Midwesterners. Participants exhibited a preference for substituting certain precooked items with fresh whole alternatives (e.g., preference for fresh strawberries served with plain oatmeal over baked oatmeal with cooked strawberries), along with an inclination toward consuming garden-obtained foods like cucumbers as snacks. The decision to consider fresh raw vegetables and other study-diet-aligned outside intakes as *compliant* allowed flexibility, potentially minimizing attrition without sacrificing scientific merit. Similarly, extensive participant interaction with the research staff enabled the recognition and implementation of allowances for the consumption of calorie-free beverages in moderation.

High consumption of UPFs has been linked to increased disease risk and gut dysbiosis, yet research in older populations remains limited. Dietary intervention studies often overlook UPF intake, a potential confounder, when assessing the effects of other dietary components, such as macronutrients, protein sources, and dietary supplements. The study revealed that participants had >53% UPF intake at baseline, which is similar to or slightly below other reports in the general population [66,67]. To our knowledge, this is the first methods-report detailing the feasibility of an RCT to restrict UPF consumption among rural older adult through a cooking-from-scratch approach. Interestingly, despite high UPF consumption, the baseline dietary intake in this older population was better aligned with the guidelines in several respects than NHANES reports among those of similar age [68]. Specifically, baseline fiber intakes were higher but still lower than recommended, and sodium intakes were lower than previous reports.

We also noted a strong general interest among participants in health, diet, and healthy study diet recipes (which were shared with participants upon request), potentially indicative of a strong consumer interest, particularly considering the plant-forward nature of the study diets. This emphasizes the broad utility of the plant-forward eating plans and menu outlined herein. However, both diets required specific, albeit minor, adjustments to facilitate a nutrient-matching study design strategy. The adjustments emphasize the importance of consumer awareness regarding potential challenges in ensuring that a plant-forward low-UPF eating plan remains nutritionally complete.

### Attrition considerations

Of note, not all RCTs involve an all-food-provided format or crossover design, which are typically more restrictive and longer in duration, respectively. One study with a design and duration similar to this trial reported 32% attrition after the trial intervention began [69]. Other RCTs also reported an attrition rate of around 30% or higher [[70], [71], [72]]. The observed rate of attrition during the intervention phase was lower in the current study than in some previous reports [31,73]. However, a higher-than-anticipated rate of attrition was observed between enrollment and the start of the intervention. The requirement of participants to take fiber supplements and increase fluid intake during run-in may have contributed to some anticipations and withdrawals, but we felt this was necessary given the dramatic increase in fiber intake during the intervention compared to habitual intakes. Attrition after the intervention begins is also an important consideration because it can potentially result in low statistical power and inconclusive outcomes. The sample size at the end of the study met the original study requirement. The observations highlight the potential for comprehensive but nonoverwhelming nutrition education at enrollment to improve participant awareness about the importance of compliance in a trial. Additionally, we found careful project planning to be a key factor for recuperating attrition loss until a desired sample size was reached.

### Strengths and limitations

This method report has several strengths and limitations. The demographic makeup of the study participants included both sexes and was characterized by a narrow age group and a predominantly Caucasian lineage due to the geographical location, having positive and negative implications. Because of lower heterogeneity, one can expect less confounding of results. However, the greater homogeneity may imply that the results are less generalizable to the greater United States population.

Additionally, as with other lifestyle interventions, participants were not blinded to treatment allocation; however, most of the data collection was performed in an investigator-blinded manner to minimize detection bias. Blinding protein sources with significant taste and texture differences, such as minimally processed meat and pulse, would pose a considerable logistical challenge; furthermore, despite the significant burden on the participants, who participated for almost 19 wk, including the 5-d run-in phase and washout, participant-experience surveys indicate that the trial was well received. The postdiet experience survey data supports that the participants made earnest efforts to adhere to the study to the best of their understanding. Adherence and intake analysis demonstrated equivalent food intake across diet arms after adjusting for various aspects of noncompliance. Compliance was commendable, particularly given the longer duration of the study and, in many cases—a lack of familiarity with pulses among the rural Midwesterners prior to the study. Although returning volunteers may have offered greater consistency owing to their prior experience, this prior experience could also mean that the level of compliance was not representative of the general population. This could have potentially influenced their behavior or responses during the study. Despite this, the methodologies presented here allow for a rigorous evaluation of health outcomes linked to distinct protein sources.

In conclusion, this trial assessed a one-to-one exchange of plant and animal protein sources within a healthy, low-UPF, macronutrient-matched, DGA diet. Lifestyle interventions that target modifiable risk factors associated with age-related conditions—such as cardiovascular disease, sarcopenia, dementia, and undernutrition—offer substantial promise for improving public health outcomes. By delaying the onset of these conditions and potentially extending health span, these interventions can significantly impact older adults at risk of health decline. The methodologies outlined in this report establish a robust framework for future real-world studies, ultimately advancing understanding and nutritional management of health issues. These data offered valuable insight into dietary and protein-specific changes that are both feasible and acceptable for the Midwestern older adult population. Importantly, we report a plant-forward DGA menu that is valuable to researchers as well as to omnivorous and vegetarian consumers. Future nutritional health research should explore additional strategies for developing and implementing effective interventions in this relatively understudied demographic, as RCTs remain the gold standard in informing public health nutrition policies.

## Author contributions

The authors’ responsibilities were as follows – MD: conceived the project, designed the research, as well as provided resources and study oversight; MD, BOdV, SV, YZ, LW, C-LL, JZ: conducted research and collected data; BOdV, SV: curated and analyzed data and generated tables and figures; MD, JLF: wrote the manuscript; MD, BOdV, SV: hold primary responsibility for the final content; and all authors: read and approved the final manuscript.

## Data availability

Data are available upon reasonable request by contacting the corresponding author.

## Declaration of AI and AI-Assisted Technologies in the Writing Process

During the preparation of this work the author(s) used [ChatGPT] in order to assist with some sentence structure and grammar. After using this tool/service, the author(s) reviewed and edited the content as needed and take(s) full responsibility for the content of the publication.

## Funding

The trials are supported by the National Pork Board (22-038), USDA Agricultural Research Service (#58-3060-2-043), and NIFA/AES (#AH831-25). The sponsors had no role in the study design, collection, analysis, and interpretation of data or writing of the report, and there were no restrictions on the submission of the report for publication.

## Conflict of interest

The authors report no conflicts of interest.

## References

[1] Coelho-Júnior H.J., Rodrigues B., Uchida M., Marzetti E. (2018). Low protein intake is associated with frailty in older adults: A systematic review and meta-analysis of observational studies. Nutrients.

[2] Bauer J., Biolo G., Cederholm T., Cesari M., Cruz-Jentoft A.J., Morley J.E. (2013). Evidence-based recommendations for optimal dietary protein intake in older people: a position paper from the PROT-AGE Study Group, J Am. Med. Dir. Assoc..

[3] Deutz N.E., Bauer J.M., Barazzoni R., Biolo G., Boirie Y., Bosy-Westphal A. (2014). Protein intake and exercise for optimal muscle function with aging: recommendations from the ESPEN Expert Group. Clin. Nutr..

[4] Landi F., Calvani R., Tosato M., Martone A.M., Ortolani E., Savera G. (2016). Anorexia of aging: risk factors, consequences, and potential treatments. Nutrients.

[5] Braden M.L., Gwin J.A., Leidy H.J. (2023). Protein source influences acute appetite and satiety but not subsequent food intake in healthy adults. J Nutr.

[6] Hall W.L., Millward D.J., Long S.J., Morgan L.M. (2003). Casein and whey exert different effects on plasma amino acid profiles, gastrointestinal hormone secretion and appetite. Br. J Nutr..

[7] Hawley A.L., Gbur E., Tacinelli A.M., Walker S., Murphy A., Burgess R. (2020). The short-term effect of whey compared with pea protein on appetite, food intake, and energy expenditure in young and older men. Curr. Dev. Nutr..

[8] Abou-Samra R., Keersmaekers L., Brienza D., Mukherjee R., Macé K. (2011). Effect of different protein sources on satiation and short-term satiety when consumed as a starter. Nutr. J..

[9] Morgan P.T., Harris D.O., Marshall R.N., Quinlan J.I., Edwards S.J., Allen S.L. (2021). Protein source and quality for skeletal muscle anabolism in Young and older adults: A systematic review and meta-analysis. J Nutr.

[10] van Vliet S., Burd N.A., van Loon L.J. (2015). The skeletal muscle anabolic response to plant- versus animal-based protein consumption. J Nutr.

[11] Zheng J., Zhu T., Yang G., Zhao L., Li F., Park Y.M. (2022). The isocaloric substitution of plant-based and animal-based protein in relation to aging-related health outcomes: A systematic review. Nutrients.

[12] Huang J., Liao L.M., Weinstein S.J., Sinha R., Graubard B.I., Albanes D. (2020). Association between plant and animal protein intake and overall and cause-specific mortality. JAMA Intern. Med..

[13] Gao R., Yang Z., Yan W., Du W., Zhou Y., Zhu F. (2022). Protein intake from different sources and cognitive decline over 9 years in community-dwelling older adults. Front Public Health.

[14] Millward D.J., Layman D.K., Tomé D., Schaafsma G. (2008). Protein quality assessment: impact of expanding understanding of protein and amino acid needs for optimal health. Am. J Clin. Nutr..

[15] Dietary protein quality evaluation in human nutrition, Report of an FAQ expert consultation (2013). FAO Food Nutr. Pap..

[16] Schaafsma G. (2012). Advantages and limitations of the protein digestibility-corrected amino acid score (PDCAAS) as a method for evaluating protein quality in human diets. Br. J Nutr..

[17] Kim I.Y., Shin Y.A., Schutzler S.E., Azhar G., Wolfe R.R., Ferrando A.A. (2018). Quality of meal protein determines anabolic response in older adults. Clin. Nutr..

[18] Park S., Church D.D., Schutzler S.E., Azhar G., Kim I.Y., Ferrando A.A. (2021). Metabolic evaluation of the dietary guidelines’ ounce equivalents of protein food sources in Young adults: A randomized controlled trial. J Nutr.

[19] Church D.D., Hirsch K.R., Park S., Kim I.Y., Gwin J.A., Pasiakos S.M. (2020). Essential amino acids and protein synthesis: insights into maximizing the muscle and whole-body response to feeding. Nutrients.

[20] Wang Y., Hill E.R., Campbell W.W., O’Connor L.E. (2022). Plant- and animal-based protein-rich foods and cardiovascular health. Curr. Atheroscler. Rep..

[21] Hudson J.L., Wang Y., Bergia R.E., Campbell W.W. (2020). Protein intake greater than the RDA differentially influences whole-body lean mass responses to purposeful catabolic and anabolic stressors: A systematic review and meta-analysis. Adv. Nutr..

[22] Ferrari L., Panaite S.A., Bertazzo A., Visioli F. (2022). Animal- and plant-based protein sources: A scoping review of human health outcomes and environmental impact. Nutrients.

[23] Clem J., Barthel B. (2021). A look at plant-based diets, Mo. Med..

[24] Hargreaves S.M., Rosenfeld D.L., Moreira A.V., Zandonadi R.P. (2023). Plant-based and vegetarian diets: an overview and definition of these dietary patterns. Eur. J Nutr..

[25] Harvard Health Publishing (2024).

[26] US Department of Agriculture, US Department of Health and Human Services (2020).

[27] Ellithorpe M.E., Takahashi B., Alumit Zeldes G., Dorrance-Hall E., Chavez M., Plasencia J. (2022). Family and cultural perceptions about meat consumption among Hispanic/Latino and White adults in the United States. Ecol. Food Nutr.

[28] Crimarco A., Springfield S., Petlura C., Streaty T., Cunanan K., Lee J. (2020). A randomized crossover trial on the effect of plant-based compared with animal-based meat on trimethylamine-N-oxide and cardiovascular disease risk factors in generally healthy adults: Study With Appetizing Plantfood-Meat Eating Alternative Trial (SWAP-MEAT). Am. J Clin. Nutr..

[29] Food and Agriculture Organization of the United Nations (1994).

[30] Mitchell D.C., Marinangeli C.P., Pigat S., Bompola F., Campbell J., Pan Y. (2021). Pulse intake improves nutrient density among US adult consumers. Nutrients.

[31] Chamberlin M.L., Wilson S.M., Gaston M.E., Kuo W.Y., Miles M.P. (2024). Twelve weeks of daily lentil consumption improves fasting cholesterol and postprandial glucose and inflammatory responses-A randomized clinical trial. Nutrients.

[32] Moravek D., Duncan A.M., VanderSluis L.B., Turkstra S.J., Rogers E.J., Wilson J.M. (2018). Carbohydrate replacement of Rice or potato with lentils reduces the postprandial glycemic response in healthy adults in an acute, randomized, crossover trial. J Nutr.

[33] Wilson S.M., Peterson E.J., Gaston M.E., Kuo W.Y., Miles M.P. (2022). Eight weeks of lentil consumption attenuates insulin resistance progression without increased gastrointestinal symptom severity: A randomized clinical trial. Nutr. Res..

[34] Rebello C.J., Beyl R.A., Greenway F.L., Atteberry K.C., Hoddy K.K., Kirwan J.P. (2022). Low-energy dense potato- and bean-based diets reduce body weight and insulin resistance: A randomized, feeding, equivalence trial, J Med. Food..

[35] Hartman T.J., Christie J., Wilson A., Ziegler T.R., Methe B., Flanders W.D. (2024). Fibre-rich Foods to Treat Obesity and Prevent Colon Cancer trial study protocol: a randomised clinical trial of fibre-rich legumes targeting the gut microbiome, metabolome and gut transit time of overweight and obese patients with a history of noncancerous adenomatous polyps. BMJ Open.

[36] Itkonen S.T., Karhu P., Pellinen T., Lehtovirta M., Kaartinen N.E., Männistö S. (2024). Effects of partial replacement of red and processed meat with non-soya legumes on bone and mineral metabolism and amino acid intakes in BeanMan randomised clinical trial. Br. J Nutr.

[37] Jenkins D.J., Kendall C.W., Augustin L.S., Mitchell S., Sahye-Pudaruth S., Blanco Mejia S. (2012). Effect of legumes as part of a low glycemic index diet on glycemic control and cardiovascular risk factors in type 2 diabetes mellitus: a randomized controlled trial. Arch. Intern. Med..

[38] Ferreira H., Vasconcelos M., Gil A.M., Pinto E. (2021). Benefits of pulse consumption on metabolism and health: A systematic review of randomized controlled trials. Crit. Rev. Food Sci. Nutr..

[39] Dhakal S., Moazzami Z., Perry C., Dey M. (2022). Effects of lean pork on microbiota and microbial-metabolite trimethylamine-N-oxide: A randomized controlled non-inferiority feeding trial based on the Dietary Guidelines for Americans. Mol. Nutr. Food Res..

[40] Gwin J.A., Carbone J.W., Rodriguez N.R., Pasiakos S.M. (2021). Physiological limitations of protein foods ounce equivalents and the underappreciated role of essential amino acid density in healthy dietary patterns. J Nutr.

[41] Braden M.L., Gwin J.A., Leidy H.J. (2024). A diet containing animal source protein as fresh, lean beef is more well liked and promotes healthier eating behavior compared with plant-based alternatives in women with overweight. Curr. Dev. Nutr..

[42] Most M.M., Ershow A.G., Clevidence B.A. (2003). An overview of methodologies, proficiencies, and training resources for controlled feeding studies. J Am. Diet Assoc..

[43] Moreira E.A., Most M., Howard J., Ravussin E. (2011). Dietary adherence to long-term controlled feeding in a calorie-restriction study in overweight men and women. Nutr. Clin. Pract..

[44] Van Horn L., Carson J.A., Appel L.J., Burke L.E., Economos C., Karmally W. (2016). Recommended dietary pattern to achieve adherence to the American Heart Association/American College of Cardiology (AHA/ACC) guidelines: A scientific statement from the American Heart Association. Circulation.

[45] Krishnan S., Lee F., Burnett D.J., Kan A., Bonnel E.L., Allen L.H. (2020). Challenges in designing and delivering diets and assessing adherence: A randomized controlled trial evaluating the 2010 Dietary Guidelines for Americans. Curr. Dev. Nutr..

[46] National Heart, Lung, and Blood Institute. DASH eating plan [cited 2024]. Available from: https://www.nhlbi.nih.gov/education/dash-eating-plan.

[47] Oktaviono Y.H., Dyah Lamara A., Saputra P.B., Arnindita J.N., Pasahari D., Saputra M.E. (2023). The roles of trimethylamine-N-oxide in atherosclerosis and its potential therapeutic aspect: A literature review. Biomol. Biomed..

[48] Stumbo P. (2008). Considerations for selecting a dietary assessment system, J Food Compost. Anal..

[49] Monteiro C.A., Cannon G., Levy R.B., Moubarac J.C., Louzada M.L., Rauber F. (2019). Ultra-processed foods: what they are and how to identify them. Public Health Nutr.

[50] Mendonça R.D., Pimenta A.M., Gea A., de la Fuente-Arrillaga C., Martinez-Gonzalez M.A., Lopes A.C. (2016). Ultraprocessed food consumption and risk of overweight and obesity: the University of Navarra Follow-Up (SUN) cohort study. Am. J Clin. Nutr..

[51] Valmorbida J.L., Baratto P.S., Leffa P.S., Sangalli C.N., Silva J.A., Vitolo M.R. (2023). Consumption of ultraprocessed food is associated with higher blood pressure among 6-year-old children from southern Brazil. Nutr. Res..

[52] Forester S.M., Jennings-Dobbs E.M., Sathar S.A., Layman D.K. (2023). Perspective: developing a nutrient-based framework for protein quality. J Nutr.

[53] Shams-White M.M., Pannucci T.E., Lerman J.L., Herrick K.A., Zimmer M., Meyers Mathieu K. (2023). Healthy eating Index-2020: review and update process to reflect the Dietary Guidelines for Americans,2020-2025. J Acad. Nutr. Diet..

[54] Martínez Steele E., Baraldi L.G., Louzada M.L., Moubarac J.C., Mozaffarian D., Monteiro C.A. (2016). Ultra-processed foods and added sugars in the US diet: evidence from a nationally representative cross-sectional study. BMJ Open.

[55] Kinoshita K., Otsuka R., Takada M., Tsukamoto-Yasui M., Nishita Y., Tange C. (2021). The association between dietary amino acid intake and cognitive decline 8 years later in Japanese community-dwelling older adults. J Nutr. Health Aging..

[56] Grajeda-Iglesias C., Aviram M. (2018). Specific amino acids affect cardiovascular diseases and atherogenesis via protection against macrophage foam cell formation: review article, Rambam. Maimonides. Med. J..

[57] Kahleova H., Fleeman R., Hlozkova A., Holubkov R., Barnard N.D. (2018). A plant-based diet in overweight individuals in a 16-week randomized clinical trial: metabolic benefits of plant protein. Nutr. Diabetes.

[58] Richardson N.E., Konon E.N., Schuster H.S., Mitchell A.T., Boyle C., Rodgers A.C. (2021). Lifelong restriction of dietary branched-chain amino acids has sex-specific benefits for frailty and lifespan in mice. Nat. Aging..

[59] Martínez-Arnau F.M., Fonfría-Vivas R., Buigues C., Castillo Y., Molina P., Hoogland A.J. (2020). Effects of leucine administration in sarcopenia: A randomized and placebo-controlled clinical trial. Nutrients.

[60] Kang Y., Kim N., Choi Y.J., Lee Y., Yun J., Park S.J. (2020). Leucine-enriched protein supplementation increases lean body mass in healthy Korean adults aged 50 years and older: A randomized, double-blind, placebo-controlled trial. Nutrients.

[61] MacArthur M.R., Mitchell S.J., Treviño-Villarreal J.H., Grondin Y., Reynolds J.S., Kip P. (2021). Total protein, not amino acid composition, differs in plant-based versus omnivorous dietary patterns and determines metabolic health effects in mice. Cell Metab..

[62] Centre for Evidence-Based Medicine (2016). https://www.cebm.net/2016/05/ocebm-levels-of-evidence/.

[63] Hébert J.R., Frongillo E.A., Adams S.A., Turner-McGrievy G.M., Hurley T.G., Miller D.R. (2016). Perspective: randomized controlled trials are not a panacea for diet-related research. Adv. Nutr..

[64] Krishnan S., Adams S.H., Allen L.H., Laugero K.D., Newman J.W., Stephensen C.B. (2018). A randomized controlled-feeding trial based on the Dietary Guidelines for Americans on cardiometabolic health indexes. Am. J Clin. Nutr..

[65] Ratnayake M., Lukas S., Brathwaite S., Neave J., Henry H. (2022). Aging in place: are we prepared? Dela. J Public Health..

[66] Aljahdali A.A., Rossato S.L., Baylin A. (2024). Ultra-processed foods consumption among a USA representative sample of middle-older adults: a cross-sectional analysis. Br. J Nutr.

[67] Cardoso B.R., Machado P., Steele E.M. (2022). Association between ultra-processed food consumption and cognitive performance in US older adults: a cross-sectional analysis of the NHANES 2011-2014. Eur. J Nutr.

[68] US Department of Agriculture (2018).

[69] Hill E.R., Wang Y., Davis E.M., Campbell W.W. (2024). Healthy dietary patterns with and without meat improved cardiometabolic disease risk factors in adults: A randomized crossover controlled feeding trial. Nutrients.

[70] Trepanowski J.F., Kroeger C.M., Barnosky A., Klempel M.C., Bhutani S., Hoddy K.K. (2017). Effect of alternate-day fasting on weight loss, weight maintenance, and cardioprotection among metabolically healthy obese adults: A randomized clinical trial. JAMA Intern. Med..

[71] Sayer R.D., Wright A.J., Chen N., Campbell W.W. (2015). Dietary Approaches to Stop Hypertension diet retains effectiveness to reduce blood pressure when lean pork is substituted for chicken and fish as the predominant source of protein. Am. J Clin. Nutr..

[72] Rundle M., Fiamoncini J., Thomas E.L., Wopereis S., Afman L.A., Brennan L. (2023). Diet-induced weight loss and phenotypic flexibility among healthy overweight adults: A randomized trial. Am. J Clin. Nutr..

[73] O’Connor L.E., Paddon-Jones D., Wright A.J., Campbell W.W. (2018). A Mediterranean-style eating pattern with lean, unprocessed red meat has cardiometabolic benefits for adults who are overweight or obese in a randomized, crossover, controlled feeding trial. Am. J Clin. Nutr..

