# Supplementary material for: Design and Implementation of the Protein-Distinct Macronutrient-Equivalent Diet (PRODMED) Study: An Eighteen-Week Randomized Crossover Feeding Trial Among Free-Living Rural Older Adults
Source: Curr Dev Nutr. 2025 Mar 24;9(5):104588. doi: 10.1016/j.cdnut.2025.104588 (PMC12032901; doi:10.1016/j.cdnut.2025.104588)
Supplement: multimedia component 1 [file mmc1.docx]

**Supplementary Material**

**Title:**

**Design and Implementation of the Protein-Distinct Macronutrient-Equivalent Diet (PRODMED) Study: An Eighteen-Week Randomized Crossover Feeding Trial Among Free-Living Rural Older Adults**

**Bruna O. de Vargas** et al.

**Table S1A.** Plant-protein diet (PPD) weekly rotating menu.

|  | **Monday** | **Tuesday** | **Wednesday** | **Thursday** | **Friday** | **Saturday** | **Sunday** |
| --- | --- | --- | --- | --- | --- | --- | --- |
| **Breakfast entrees** | Quinoa cooked with fruits and pecans | Blueberry coffee cake | Chocolate- strawberry baked oatmeal (old fashioned oats) | Carrot cake baked oatmeal (old fashioned oats) | Farina cooked with vegetables | Pancake with banana and maple syrup | Banana bread baked oatmeal (old fashioned oats) |
| **Lunch entrees**  **(pulses and**  **other main ingredients)** | Fried rice (ground plant meat, chickpea rice, brown rice, green pea, carrot, green beans,  pea protein powder) | Enchilada spaghetti squash (black beans, squash, pepper, onion, olive, cheddar cheese, pea protein powder) | Fajita bowl  (black beans, mushroom, bell peppers, onion, quinoa, pea protein powder) | Maple Chipotle bowl (ground plant meat, chickpea rice, maple syrup, spinach, butternut squash, brussels sprouts, pea protein powder) | Butternut squash risotto (ground plant meat, onion, arborio rice, butternut squash, mushroom) + boiled green split pea | Shepherd’s pie (red lentil, potato, carrot, green pea, celery, onion, tomato, mushroom, pea protein powder) + boiled green split pea | Alfredo pasta with fruit cocktail (red lentil penne, ground plant meat, parmesan cheese, cream, broccoli) |
| **Dinner entrees**  **(pulses and**  **other main ingredients)** | Cacciatore  (red lentil, brown rice, tomato, bell pepper, carrot, pea protein powder) | Lemon risotto (arborio rice, asparagus, green onion, spinach, mushroom, broccoli, pea protein powder) | Stew (chickpea, bell pepper, carrot, tomato, cauliflower, pea protein powder) | Overloaded baked potato  (red lentil, potato, cheese, corn, bell pepper, green onion, pea protein powder) | Pesto pita (chickpea, bell pepper, zucchini, onion, basil pesto, pita, pea protein powder) | Pesto penne with breadstick (red lentil penne, tomato, basil pesto, walnut, parmesan cheese, chickpea, pea protein powder) | Lo mein (chickpea rice, green pea, bell pepper, onion, broccoli, green bean, mushroom, carrot, corn, pea protein powder) |
| **Snacks and sides** | Egg, yogurt, corn puff, white chocolate sq. | Gogurt, egg, milk, yogurt, potato crisp | Fruit cup, yogurt, potato crisp | Fruit cup, yogurt,  corn puffs | Egg, Gogurt, corn puffs | Egg | Fruit cup, milk, potato crisp |
| **Beverages** | ~64 oz per day including water, tea, and black coffee (other zero-calorie beverages in moderation) | | | | | | |

**Table S1B.** Meat-protein diet **(**MPD) weekly rotating menu.

|  | **Monday** | **Tuesday** | **Wednesday** | **Thursday** | **Friday** | **Saturday** | **Sunday** |
| --- | --- | --- | --- | --- | --- | --- | --- |
| **Breakfast entrees** | Quinoa cooked with fruits and pecans | Blueberry coffee cake | Chocolate- strawberry baked oatmeal (old fashioned oats) | Carrot cake baked oatmeal (old fashioned oats) | Farina cooked with vegetables | Pancake with banana and maple syrup | Banana bread  baked oatmeal (old fashioned oats) |
| **Lunch entrees**  **(main ingredients)** | Fried rice (pork, brown rice, green pea, carrot, green beans) | Enchilada spaghetti squash (pork, olive, squash, pepper, onion,  cheddar cheese, corn) | Fajita bowl (pork, bell pepper, onion, quinoa, corn, spinach) | Maple Chipotle (pork, brown rice, spinach, maple syrup, butternut squash, brussels sprouts) | Butternut squash risotto (pork, arborio rice, butternut squash, spinach, onion)  + steamed  broccoli | Shepherd’s pie  (pork, potato, carrot, green pea, celery, onion, mushroom, tomato) + steamed green beans | Alfredo pasta with fruit cocktail (pork, whole wheat penne, parmesan cheese, cream, broccoli) + stir fried green peas |
| **Dinner entrees (main ingredients)** | Cacciatore  (pork, brown rice, tomato, bell pepper, carrot)  + steamed green beans | Lemon risotto (pork, arborio rice, asparagus, green onion, mushroom, spinach, broccoli, onion) + steamed broccoli | Stew (pork, bell pepper, carrot, cauliflower, tomato, corn) + Stir-fried green peas | Overloaded baked potato (pork, potato, cheese, corn, bell pepper, green onion) + steamed green beans | Pesto pita (pork, bell pepper, zucchini, onion, basil pesto, whole wheat pita) | Pesto penne with breadstick (pork, whole wheat penne, tomato, basil pesto, walnut, parmesan cheese) | Lo mein (pork, whole grain linguine, green pea, bell pepper, onion, broccoli, green beans, mushroom, carrot, corn) |
| **Snacks and sides** | Orange juice, dark chocolate sq, potato crisp | Orange juice, corn puffs | Fruit cup, potato crisp, orange juice | Fruit cup, potato crisp | Egg, orange juice, corn puffs | Egg, granola bar, potato crisp | Fruit cup, corn puffs |
| **Beverages** | ~ 64 oz per day including water, tea, and black coffee (other zero calorie beverages in moderation) | | | | | | |

Condiments, herbs, spices and seasonings used in recipes as needed:

Barbecue sauce, black olive, chili, chipotle peppers in freshly prepared adobo sauce, cilantro, cocoa powder, coconut amino, dijon mustard, dry basil, no sodium baking powder, soluble fiber added to some pork meals to increase fiber intake, ginger, ground cinnamon, ground cumin, honey, Italian seasoning, jalapeno, lemon juice, lemon zest, onion, paprika, red pepper flakes, dried rosemary, table salt, seeds (chia, coriander, flaxseed), taco seasoning, thyme, unsalted butter, vanilla extract, vegetable oil, vinegar (apple cider, balsamic, red wine, rice), yeast flakes.

Small portions of zero-calorie flavored syrups were provided upon request to use with unflavored/unsweetened yogurts.

All the fruit cups and orange juice are 100% with no added sugar.


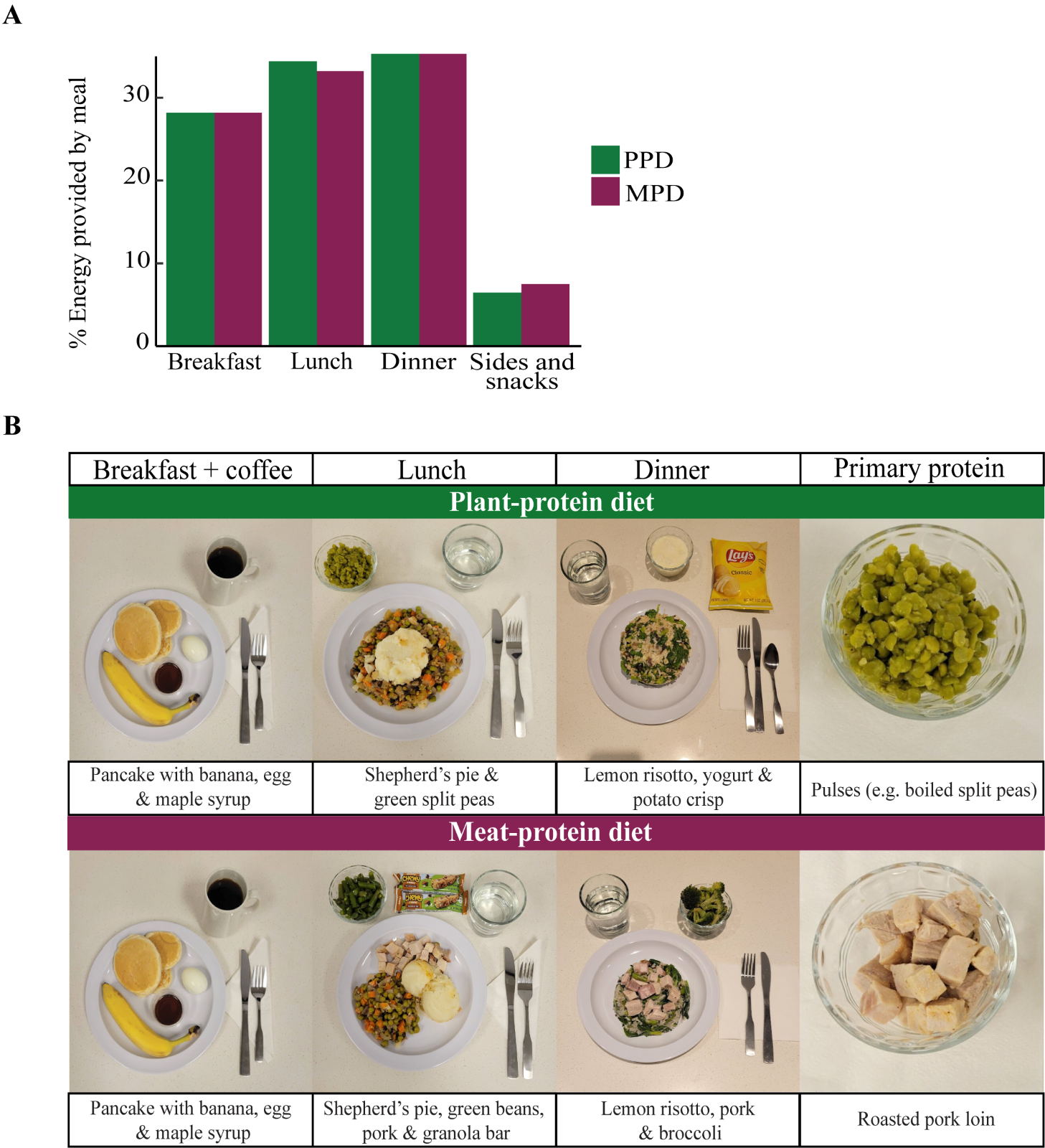


**Figure S1. A:** The average per day energy contribution from different meals across two study arms, all *p*>0.05 between study arms. **B:** Example study meals. MPD: Meat-protein diet; PPD: Plant-protein diet.

**Table S2.** Non-essential amino acid content between baseline intake and provided intervention diets.

| **Amino acid** | **Baseline** | **PPD**^1^ | **PPS**^1^ | **MPD**^1^ | **MPS**^1^ |
| --- | --- | --- | --- | --- | --- |
| Alanine (mg/d) | 2944.2 | 2508.6 | 1132.2 | 3815.3 | 2486.9 |
| Arginine (mg/d) | 3638.5 | 3632.6 | 2107.4 | 4502.7 | 2818.8 |
| Aspartic Acid (mg/d) | 5670.1 | 6000.2 | 2833.7 | 7328.2 | 4144.8 |
| Glutamic Acid (mg/d) | 11876.7 | 10251.5 | 4072.7 | 12574.9 | 6762.8 |
| Glycine (mg/d) | 2622.7 | 2006.5 | 1028.6 | 2939.2 | 1908.9 |
| Proline (mg/d) | 3963.7 | 3302.3 | 1044.6 | 3562.6 | 1712.9 |
| Serine (mg/d) | 2886.6 | 2794.4 | 1154.0 | 3218.1 | 1831.1 |

^1^MPD, Meat-protein diet; MPS, Meat-protein source; PPD, Plant-protein diet*.* PPS, Plant-protein source. ^2^Data are presented as means.

^3^Glutamine and asparagine values are not reported by Nutritionist Pro.

**Box S1.** Instructions on safe storage and reheating provided to study participants.

| **Safe storage and reheating instructions:**   - **Storage:**   - Keep the meal refrigerated if consuming within a few days.   - If not consuming immediately, freeze by the "Use or Freeze By" date indicated on the label. - **Reheating (from refrigerated):**   - Microwave**:** The bowl provided is microwave safe. Remove the lid and heat for 1–2 minutes or until the meal reaches an internal temperature of 165°F (74°C).   - Oven: Preheat to 350°F (177°C), transfer to an oven-safe dish, and heat for 10–15 minutes or until thoroughly warmed. - Do not reheat more than once. - Ensure food is piping hot before consuming. - Follow specific ingredient guidelines if provided on the label. - Note down food intake on the provided checklist. |
| --- |

**Table S3.** Proposed key considerations when designing meal provision in randomized feeding-controlled trials.

| **Category** | **Considerations** |
| --- | --- |
| Cultural considerations | Account for cultural influences and regional food preferences.  Use local flavors and ingredients when possible. |
| Meal format | Provide pre-portioned, ready-to-heat meals to minimize preparation time and ensure standardized intake. |
| Menu adherence control | Incorporate a menu adherence checklist for participants to record how much of the study food they consume and any intake of outside foods.  Add color-coded labeling for meal identification (e.g., Saturday lunch).  Include easy-to-follow heating instructions. |
| Meal variety | Ensure variety to prevent meal fatigue while maintaining logistical feasibility. |
| Perishable foods | Depending on logistical conditions, minimize fresh fruits and salad greens, substituting them with alternatives included in the recipes. |
| Nutrition education | Offer clear and concise education to enhance compliance, including dietary restrictions and study-specific requirements. |
| Nutritional software | Ensure the software includes a comprehensive, validated nutrient database, customizable options, and meal planning features with all required nutritional information.  Easy to understand output format. |
| Flexibility | Consider flexible meal dine-ins and pick-ups or delivery to accommodate participant schedules.  Include provision for calorie-intake adjustments if feasible aligned with study goals. |
